# Social Attention Deficits in Children With Autism Spectrum Disorder: Task Dependence of Objects vs. Faces Observation Bias

**DOI:** 10.3389/fpsyt.2021.640599

**Published:** 2021-03-22

**Authors:** Susana Mouga, João Castelhano, Cátia Café, Daniela Sousa, Frederico Duque, Guiomar Oliveira, Miguel Castelo-Branco

**Affiliations:** ^1^CIBIT - Coimbra Institute for Biomedical Imaging and Translational Research, University of Coimbra, Coimbra, Portugal; ^2^ICNAS - Institute of Nuclear Sciences Applied to Health, University of Coimbra, Coimbra, Portugal; ^3^CNC.IBILI – Institute for Biomedical Imaging and Life Sciences, Faculty of Medicine, University of Coimbra, Coimbra, Portugal; ^4^Neurodevelopmental and Autism Unit From Child Developmental Centre, Hospital Pediátrico, Centro Hospitalar e Universitário de Coimbra, Coimbra, Portugal; ^5^Centro de Investigação e Formação Clínica, Hospital Pediátrico, Centro Hospitalar e Universitário de Coimbra, Coimbra, Portugal; ^6^University Clinic of Pediatrics, Faculty of Medicine, University of Coimbra, Coimbra, Portugal; ^7^Faculty of Medicine, University of Coimbra, Coimbra, Portugal

**Keywords:** autism spectrum disorder, social attention, eye-tracking, attentional bias, autism diagnostic observation schedule

## Abstract

Social attention deficits represent a central impairment of patients suffering from autism spectrum disorder (ASD), but the nature of such deficits remains controversial. We compared visual attention regarding social (faces) vs. non-social stimuli (objects), in an ecological diagnostic context, in 46 children and adolescents divided in two groups: ASD (*N* = 23) and typical neurodevelopment (TD) (*N* = 23), matched for chronological age and intellectual performance. Eye-tracking measures of visual scanning, while exploring and describing scenes from three different tasks from the Autism Diagnostic Observation Schedule (ADOS), were analyzed: “Description of a Picture,” “Cartoons,” and “Telling a Story from a Book.” Our analyses revealed a three-way interaction between Group, Task, and Social vs. Object Stimuli. We found a striking main effect of group and a task dependence of attentional allocation: while the TD attended first and longer to faces, ASD participants became similar to TD when they were asked to look at pictures while telling a story. Our results suggest that social attention allocation is task dependent, raising the question whether spontaneous attention deficits can be rescued by guiding goal-directed actions.

## Introduction

Autism spectrum disorder (ASD) is an early-onset neurodevelopmental disorder marked by the specificity of significant impairments in social interaction and communication, restricted interests, and the presence of repetitive and stereotyped behaviors (1). Social deficits in other domains include deviations in basic attentional processes, impairments in attention to faces or social stimuli across the lifespan, as well as attention during social exchanges; for a review, see (2–5).

The ability to direct the attention to social stimuli is present and evident in typically developing children from early infancy onwards (6–8). The attention to faces serves as a window into individuals' emotional and intentional states, providing critical information for social, cognitive, and communicative development and functioning (9–14). It has been hypothesized that deficits in social attention present in ASD, such as reduced attention to social stimuli as a whole or atypical allocation of attention to social stimuli, are the cause of a compromised social functioning. Such deficits might lead to reduced social processing, loss of information necessary for the development of adaptive social functioning (15, 16), and difficulties in the interpretation of emotional information (17, 18).

Eye-tracking studies in ASD showed a correlation between reduced attention to social stimuli and behavioral measures (19–22). Klin et al. (21) showed, early on, that adolescents with ASD spent significantly less time attending to people when watching a segment of a movie and more time attending to the objects and the background of the scene. Deficits in social attention were thereafter replicated: (i) when looking at pictures of social scenes, participants with ASD spent less time attending to faces (23); (ii) ASD children showed no difference in the time looking at people or objects, unlike in typical neurodevelopment (TD) (24); and (iii) ASD attended less to the activities of others and focused more on the background objects (22).

Atypical imbalance in the attention for social (e.g., videos of playing children) vs. non-social stimuli (repeated geometrical patterns) in ASD was reported in a large-cohort study (25). A meta-analysis on gaze patterns (26) suggested the presence in ASD of a problem in selecting socially relevant vs. irrelevant information.

Other studies, however, do not confirm this hypothesis. Kemner et al. (27) found that the fixation times on face drawings embedded in an assortment of distractors of both children with ASD and TD were similar. Parish-Morris et al. (28) found that ASD and TD children and adolescents did not differ in the attention toward movies of faces as opposed to objects. In a study focused on magic, Kuhn et al. (29) found that ASD individuals were more susceptible to magic tricks, relying on sensitivity to social cues, than TD controls, contrary to their expectation. They found no between-group differences in fixation time on the magician's face and eyes. These studies suggest that the type of context and task may be relevant to disclose differences in social attentional allocation.

Several studies with infants suggest that innate or early-emerging attentional biases for faces or complex social scenes may be intact within the first months of life in infants who later develop ASD (30, 31) in line with negative results from behavioral studies in early infancy (32).

Other important aspect to consider in the study of social attention with eye tracking in ASD is the ecological and task relevance of the stimuli. Static stimuli have been associated with no group differences, which might indicate that they are not optimally sensitive. Adding to this information, it has been suggested that scenes depicting ecological social interactions are the ones that evoke robust social responses (33–35).

So far, no consensus has been reached on whether social attention is fundamentally reduced or absent in individuals with ASD. We hypothesize that the role of type of stimuli and task is critical. This may explain why a large number of studies show significantly diminished attention to social information in ASD compared to TD controls (21–23, 36–39), while many others show no differences (27–29, 40–44). Given this discrepancy, it is important to understand whether the putative social attention deficits are task and stimuli dependent.

We previously showed that task and context are determinant for perceptual performance in ASD, only showing coherence loss in task conditions favoring local attentional analysis (45). The same might hold for the attentional bias that characterizes this population. In this study, we extended this prior work by comparing attention allocation to social vs. non-social stimuli in three tasks in ASD and TD children and adolescents. We used a paradigm based on stimuli from the Autism Diagnostic Observation Schedule (ADOS) (46), a diagnostic tool that we used as a routine in the diagnostic procedure, to try to see if it can discriminate between ASD and TD children in what concerns to visual social attention and corroborate the attentional bias claim. We hypothesized that ASD children differ from TD children in visual attention to social stimuli and, in particular, demonstrate less looking toward faces than TD children.

## Methods

### Participants

The study comprised two groups of participants: the experimental, composed by individuals with ASD without intellectual disability (1), and the control, composed by individuals with typical neurodevelopment (TD). A total of 46 children and adolescents were enrolled in the study: 23 for the ASD group (22 male, 1 female; mean age = 13 years and 1 month, minimum age = 8 years and 10 months, maximum age = 17 years and 4 months) and 23 for the control group (21 Male, 2 female; mean age = 13 years and 5 months, minimum age = 8 years and 5 months, maximum age = 17 years and 8 months). Sample sizes were based on previously established effect sizes from other studies using eye tracking (23). Groups were matched by chronological age, gender, and performance intelligence quotient (PIQ) (47). Analyses were conducted to ensure that participants were matched with respect to chronological age, gender, handedness, and PIQ in both groups (*T*-test, *p* > 0.05). Further group characterization details can be found in Table 1.

**Table 1:** Characterization of the clinical and control groups.

	**ASD**	**TD**	
	**Mean (SE)**	**Mean (SE)**	***T* tests**
*N*	23	23	
Gender (M/F)	22/1	21/2	*p* > 0.05
CA (months)	156.8 (4.9)	160.5 (6.4)	*p* > 0.05
FSIQ	99.2 (3.0)	124.8 (4.1)	
VIQ	96.3 (2.7)	123.9 (4.1)	
PIQ	104.0 (3.2)	112.1 (3.6)	*p* > 0.05
ADI-R RSI	15.7 (1.1)	–	
ADI-R L/C	9.4 (0.8)	–	
ADI-R RB/I	5.1 (0.6)	–	
ADOS COM	4.6 (0.3)	–	
ADOS SI	8.0 (0.5)	–	
ADOS Total	12.6 (0.7)	–	

*ASD, Autism Spectrum Disorder group; TD, typical neurodevelopment group; SE, standard error of the mean; M, male; F, female; CA, chronological age; FSIQ, Full-Scale Intelligence quotient (IQ); VIQ, Verbal IQ; PIQ, Performance IQ; ADI-R RSI, ADI-R Reciprocal Social Interactions; ADI-R L/C, Autism Diagnostic Interview–Revised Language/Communication; RB/I, ADI-R Repetitive Behaviors/Interests; ADOS COM, ADOS Communication; ADOS SI, ADOS Social Interaction. T-tests; p > 0.05*.

ASD participants were recruited from a sample from the Neurodevelopmental and Autism Unit, Child Developmental Center, Pediatric Hospital, Centro Hospitalar e Universitário de Coimbra, Portugal. ASD diagnosis was assigned on the basis of the gold standard instruments: parental or caregiver interview, Autism Diagnostic Interview–Revised (ADI-R) (48); direct structured proband assessment, ADOS (46); and clinical examination performed by an experienced neurodevelopmental pediatrician, based on the current diagnostic criteria for autism spectrum disorder from the Diagnostic and Statistical Manual of Mental Disorders 5, DSM-5 (1). All ASD patients had positive results in the ADI-R and ADOS for autism or ASD and met the criteria for ASD from the DSM-5. A comprehensive medical observation excluded associated medical condition such as epilepsy, neurocutaneous or other genetic syndromes, or other usual comorbidities in ASD samples. TD participants were recruited from local schools and from our volunteers' database.

The exclusion criteria for the children who participated in this study were evaluated through an extensive anamnesis carried out with the parents or caregivers. They included neurological, neurodevelopmental, and genetic diseases, brain lesions, sensory, auditory, motor deficits, and neurodevelopmental milestones. Additionally, the parents of TD participants completed the Social Communication Questionnaire and Social Responsiveness Scale to ensure the exclusion of ASD symptomatology. TD participants with history of ASD symptomatology, other neurodevelopmental and neurological disorders, as well as other diseases mentioned above were excluded.

Both groups underwent an exhaustive neuropsychological evaluation and an assessment of the IQ to exclude intellectual disabilities [all participants had a Full-Scale IQ (FSIQ) > 70]. To be included in this study, participants also had to be able to read, describe pictures, and also remain still during the task. This increased the age of the participants able to be included, despite the efforts to recruit younger subjects.

### Apparatus

Eye movements were measured with a remote binocular eye tracking (SMI RED) system (SMI-SensoMotoric Instruments, Germany), with a sampling rate of 250 Hz. The tracker has a reported gaze position accuracy of 0.4° and a spatial resolution of 0.05. The participants sat ~between 60 and 70 cm away from a 22-in flat screen with a resolution of 1,680 × 1,050 pixels. The system compensates for head movements within a 50 cm × 30 cm (at 65 cm distance), allowing the participants to look at the screen in a naturalistic manner. A 9-point calibration procedure with a fixation cross was performed before each task. The children were instructed to fixate on the cross. After the calibration, there was a validation trial to ensure the precision of the data collection. The calibration process was repeated when necessary until both eyes achieved good mapping on all nine test positions (tracking error smaller than 1° visual angle).

### Visual Stimuli and Task

The experimental protocol was created and implemented through SensoMotoric Instruments Experiment Center Version 3.2 (SMI-SensoMotoric Instruments, Germany). It was composed of three types of tasks, which integrated visual stimuli adapted from the ADOS (46). The ADOS is a semistructured, examiner's dependent, tool to assess communication, social interaction, and imagination. It allows to diagnose autism spectrum disorder (ASD) across ages, neurodevelopmental levels, and language skills. The tasks from ADOS adapted were “Description of a Picture,” “Cartoons,” and “Telling a Story from a Book.” In the “Description of a Picture” task, the participant was asked to look at a scene and tell what she/he sees. In the “Cartoons” and “Telling a story from a Book” tasks, they are asked to tell a story from the images that are presented one at a time as if they were really narrating a book. Therefore, they were instructed to tell the researcher when they want to move to the following page. In total, they were 25 visual stimuli, representing social scenes, displayed on a screen.

**Figure 1:**
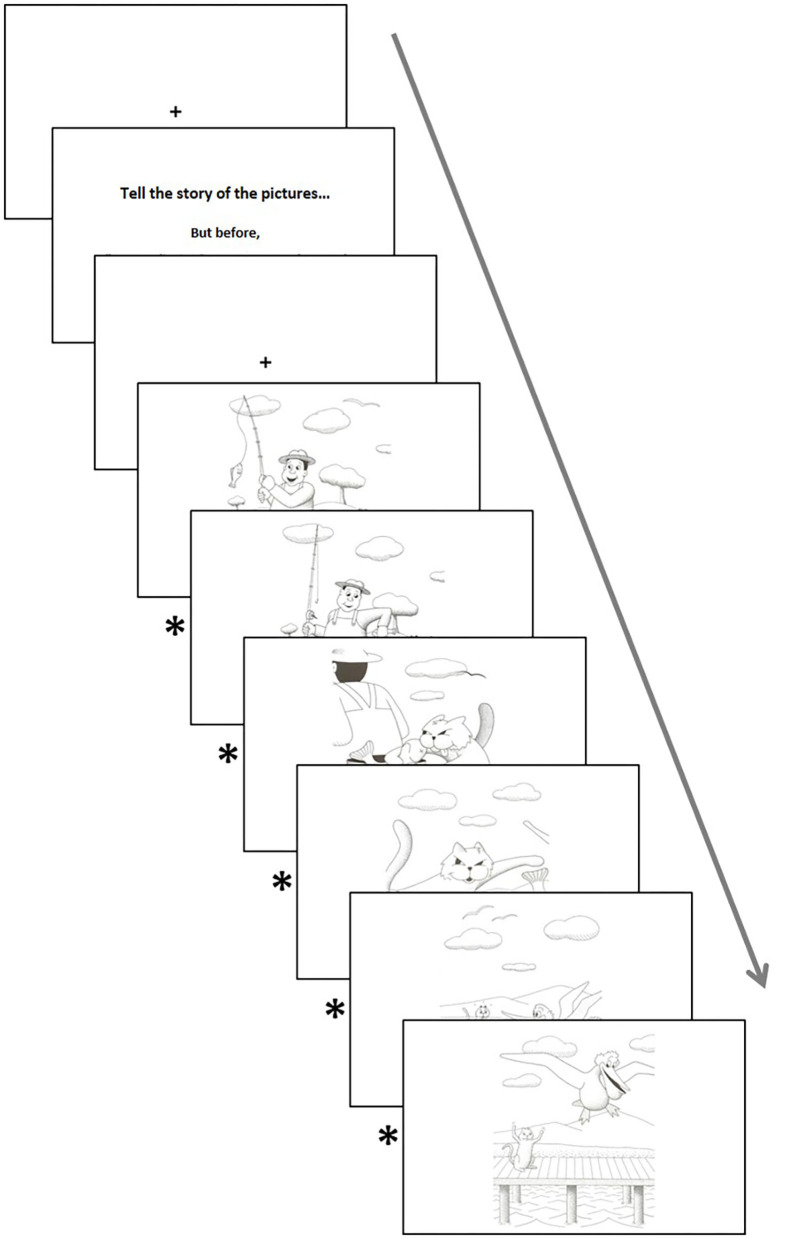
Acquisition protocol of the task “Cartoons.” *Between each image is always presented a fixation cross (signaled with an asterisk in the schematic representation of the task) to ensure that the exploration of the image begins at the same point.

Participants were allowed to move forward to the next stimulus as soon as they had described what they were seeing and told the experimenter they wanted to move to the next picture. This choice was made instead of using a button to prevent subjects' fatigue, or boredom, and a subsequent attention decrease that may lead to just pressing the button without the description. To increase subjects' attention, involvement, and motivation, and to assure that the exploration of the image begins at the same point, between each image, a fixation cross was always presented. This fixation cross disappeared once the participant's gaze was detected to be within the embedded area of interest (AOI) in it. Figure 1 illustrates the experimental procedure. There was no time constraint in each picture.

### Eye Tracking Recordings and Analysis

**Figure 2:**
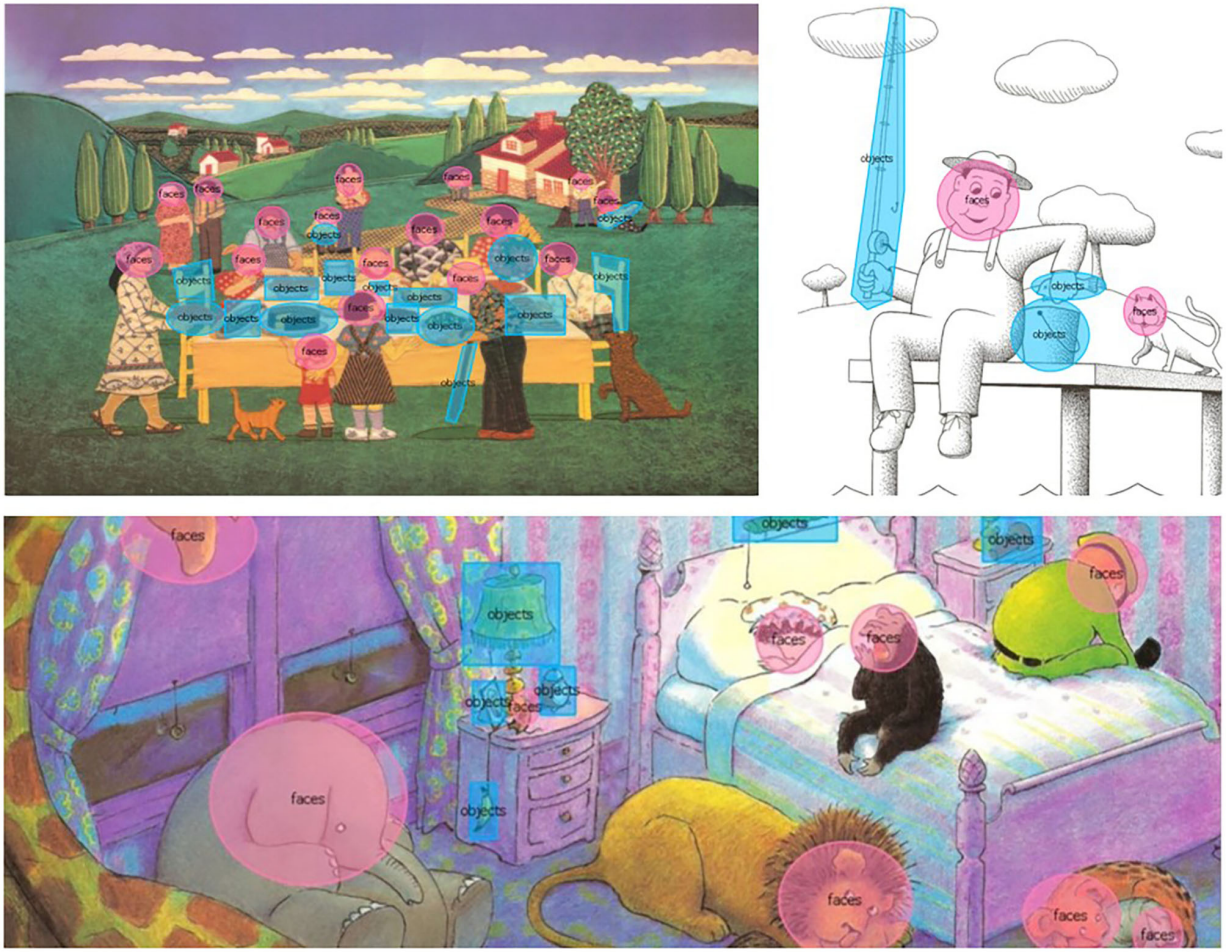
Example of the different areas of interest defined.

Eye movement data were recorded with iViewX™ 2.7 and analyzed offline with BeGaze™ 3.7 software where different AOIs were defined in a semiautomatic procedure: “faces” and “objects” (Figure 2).

We considered the following gaze metrics (defined in accordance with the manual of the BeGaze™ 3.7 software): Entry Time (ET), Dwell Time Percentage (DT%), Net Dwell Time Percentage (NDT%), Normalized Dwell (ms/coverage) (NormD), First Fixation Duration (FFD), Fixation Time Percentage (FT%), Average Fixation Duration (AFD), and Revisits. Entry Time expresses the average interval in milliseconds (ms) from the presentation of the visual stimuli (start of the trial) to the first gaze fixation on each area of interest (AOI). The Dwell Time Percentage (DT%) consists of the percentage of the sum of durations from all fixations and saccades (between the first and last fixations within the AOI) that hit the AOI (in ms), divided by the total stimulus duration. NDT% represents the percentage of the sum of sample durations for all gaze data samples that hit the AOI, divided by the total stimulus duration. NormD is the ratio between the DT and the AOI coverage, where coverage is the AOI size (measured in pixel) in comparison to stimulus size, thus representing a percentage of the number of pixel (px). It represents a more reliable measure to understand attention distribution patterns since it adjusts the duration that a subject spent to process an item relative to its surface in the display. FFD represents the duration (in ms) of the first fixation to hit the AOI. FT% consists of the percentage of the sum of the fixation durations inside the AOI, divided by the total stimulus duration. AFD is the total duration (ms) of all fixations divided by the number of fixations inside the AOI. A longer fixation duration is often associated with a deeper and more effortful cognitive processing. Revisits represent the number of time subjects visit an AOI.

### Data Analysis and Statistics

Initially, we conducted a descriptive statistics analysis in order to characterize the sample.

Eye-position data were analyzed with a standard AOI approach. Eye-tracking data were preprocessed using the SMI software, BeGaze Version 3.7 (SMI-SensoMotoric Instruments, Germany), which uses a dispersion-based algorithm for detecting fixations. The minimum fixation duration was 80 ms, and the maximum dispersion value was 100 pixels. Different aspects of eye movements were assessed. We included seven dependent variables in our eye-tracking analyses: ET, NDT%, NormD, FFD, FT%, AFD, and Revisits.

One participant in the TD group was excluded from analysis due to problems with eye-tracking data collection in tasks Picture and Book. In total, there were, therefore, valid data for 23 ASD and 22 TD participants in the Picture and Book Tasks and for 23 ASD and 23 TD participants in the Cartoons Task.

Multivariate analysis of variance (MANOVA) with a three-way interaction was used to evaluate differences in the eye-tracking measures by group, task, and AOI type (faces or objects). The goal of the three-way MANOVA was to understand if there was an interaction effect for group, AOI type, and task in the eye-tracking measures. Follow-up univariate three-way ANOVAs were run for each dependent variable. In the dependent variables with statistically significant interaction effects, simple two-way interactions and main effect of group were run. In the statistically significant main effect of group, pairwise comparisons were run with a Bonferroni adjustment applied. Effect sizes (partial η^2^ for *F* statistics and Cohen's *d* for Bonferroni) are reported with *p*-values for significant main effects, interactions, and pairwise comparisons. Our MANOVA reached a power of 92%.

All statistical analysis was completed with the support of the version for Microsoft Windows® of the Statistical Package for Social Sciences, version 26 (SPSS®, Chicago, IL, USA). A significance level of 0.05 was adopted.

### Ethics Statement

All the procedures in this study were reviewed and approved by the ethics committees from the Faculty of Medicine from University of Coimbra, Portugal (CE-11/2013) and the Centro Hospitalar e Universitário de Coimbra, Portugal (CHUC-102-13) and was conducted in accordance with the 1964 Helsinki declaration and its later amendments or comparable ethical standards. Written informed consent was obtained from the parents/guardians of all participants. Children and adolescents also gave oral informed consent.

## Results

A three-way MANOVA was run with seven eye-tracking measures (ET, NDT%, NormD, FFD, FT%, AFD, and Revisits) as dependent variables and independent variables: Group (ASD and TD), Task (Picture, Cartoons, and Book), and AOI (faces and objects). There was a statistically significant three-way interaction between Group, Task, and AOI in all our dependent variables together, Pillai's Trace = 0.092; *F*_(2, 260)_ = 1.764, *p* = 0.041, partial η^2^ = 0.046.

**Figure 3:**
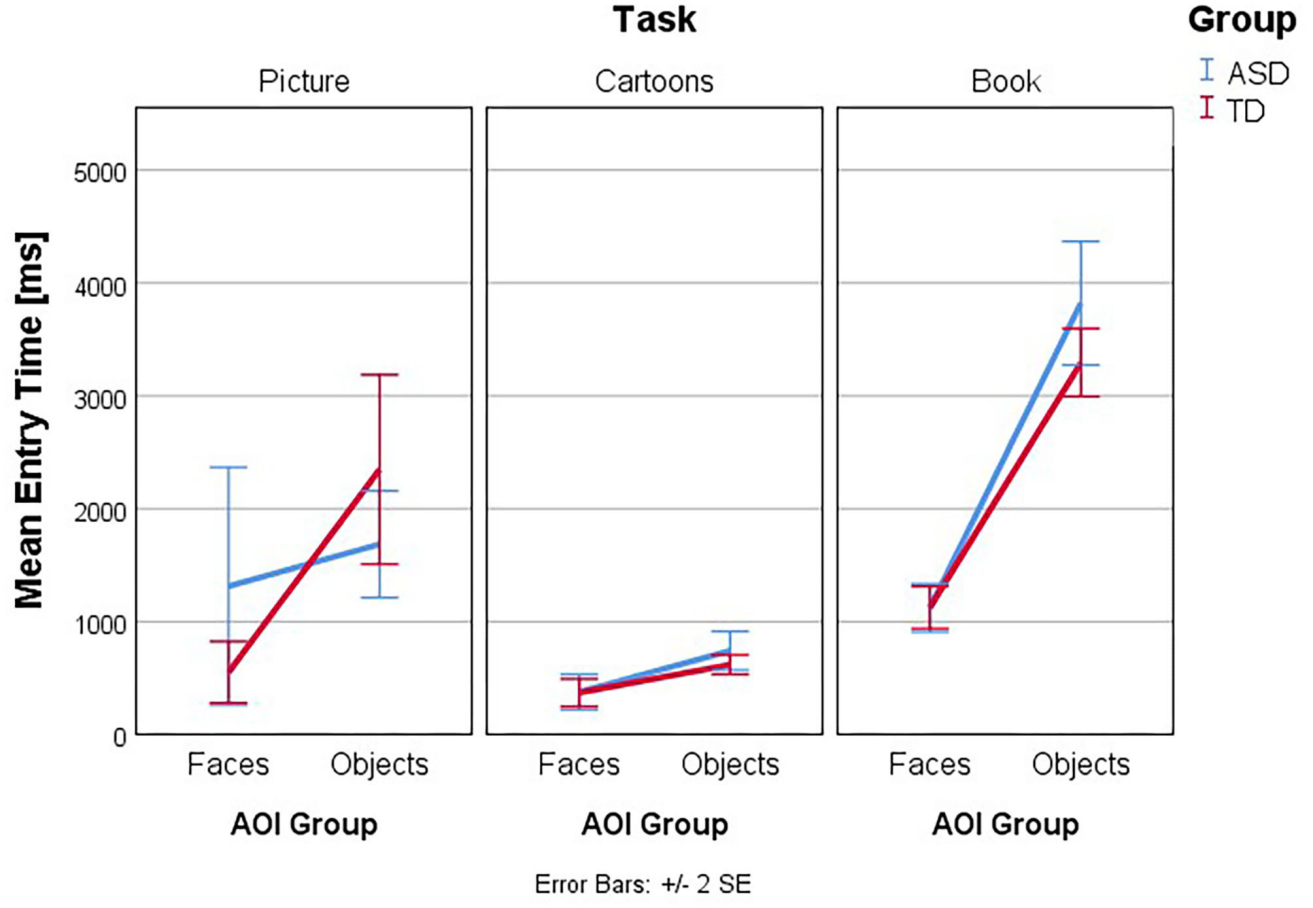
Interaction effects between group, task, and type of AOI. Mean Entry Time (ms): interaction plot Group × Task × AOI. Mean Entry Time is shown for the AOI group Faces and Objects, plotted by Group (ASD and TD) in the three tasks. Lower numbers indicate the first AOI to have the first gaze fixation.

**Figure 4:**
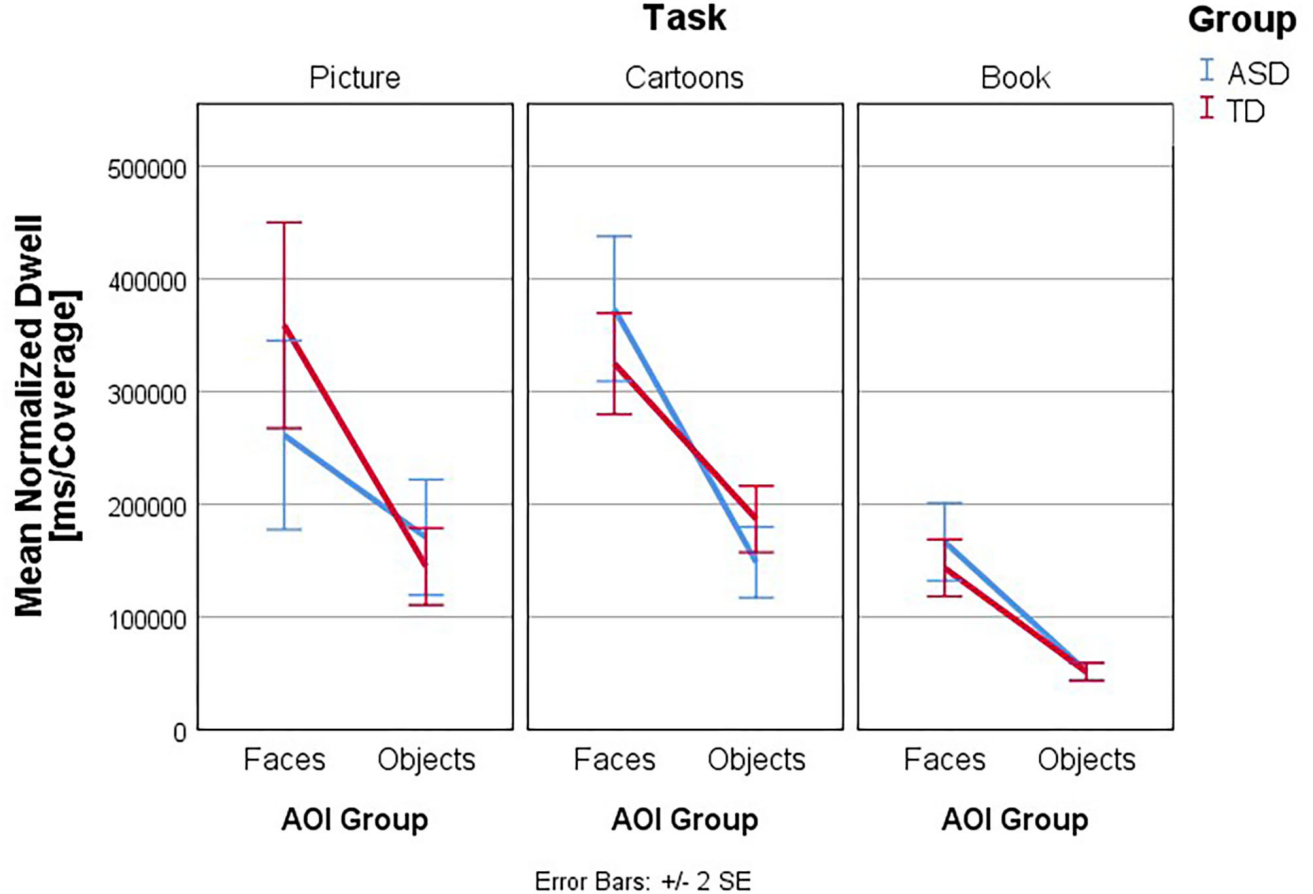
Interaction effects between group, task, and type of AOI. Mean Normalized Dwell (ms/coverage): interaction plot Group × Task × AOI. Mean Normalized Dwell is shown for the AOI group Faces and Objects, plotted by Group (ASD and TD) in the three tasks. Higher numbers indicate more time spent within the AOI, normalized by the AOI size in comparison to stimulus size, thus more time the subject spent to process the stimuli.

**Figure 5:**
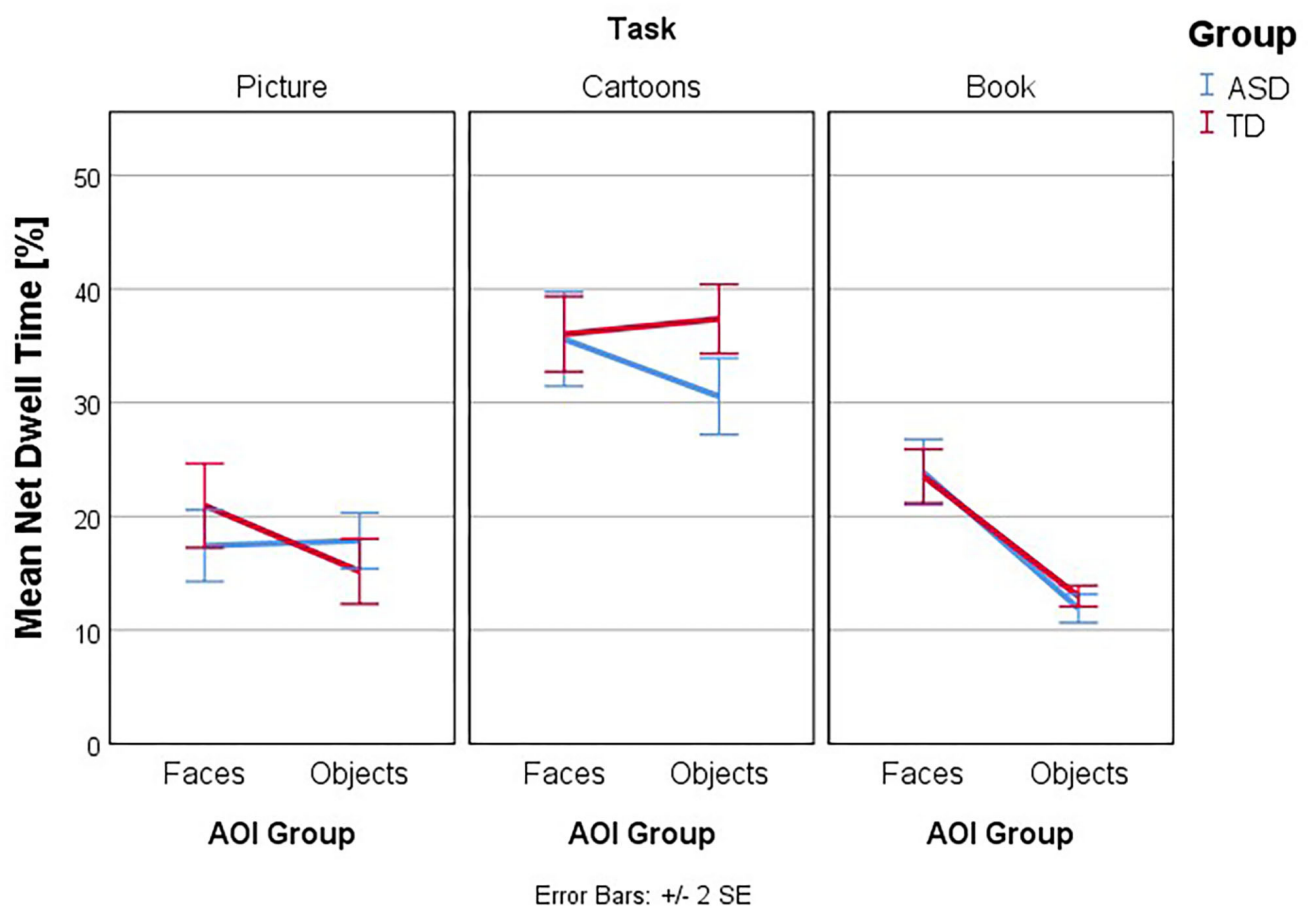
Interaction effects between group, task, and type of AOI. Mean Net Dwell Time (%): interaction plot Group × Task × AOI. Mean Net Dwell Time (%) is shown for the AOI group Faces and Objects, plotted by Group (ASD and TD) in the three tasks. Higher numbers indicate more time spent within the AOI.

**Figure 6:**
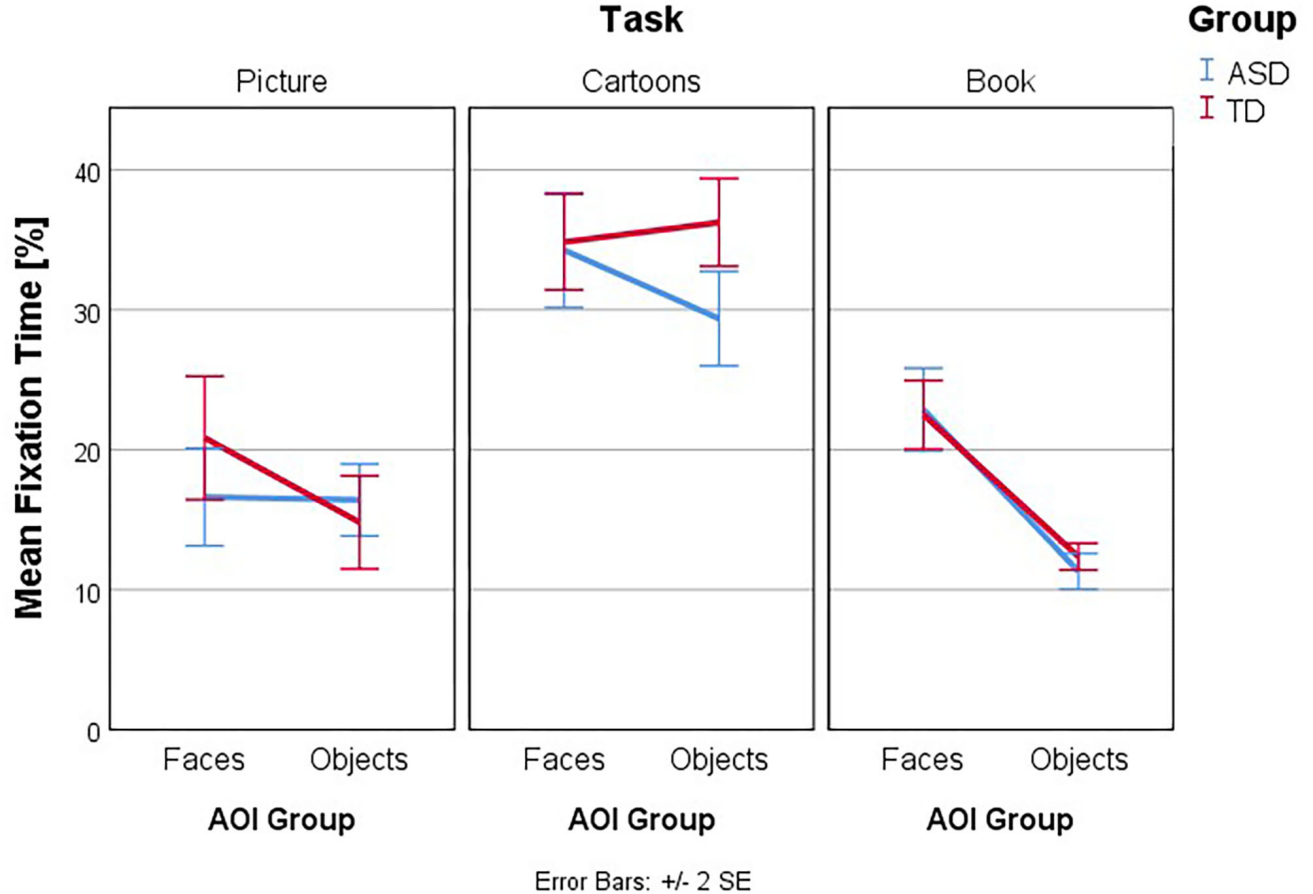
Interaction effects between group, task, and type of AOI. Mean Fixation Time (%): interaction plot Group × Task × AOI. Mean Fixation Time is shown for the AOI group Faces and Objects, plotted by Group (ASD and TD) in the three tasks. Higher numbers indicate more time spent within the AOI.

Follow-up univariate three-way ANOVAs were run for each dependent variable. These showed a statistically significant three-way interaction effect between group, task, and AOI for ET [*F*_(2, 260)_ = 4.763, *p* = 0.009, partial η^2^ = 0.035], NormD [*F*_(2, 260)_ = 4.805, *p* = 0.009, partial η^2^ = 0.036], NDT% [*F*_(2, 260)_ = 4.693, *p* = 0.010, partial η^2^ = 0.035], and FT% [*F*_(2, 260)_ = 3.81, *p* = 0.023, partial η^2^ = 0.029] but not for FFD [*F*_(2, 260)_ = 0.59, *p* =.556, partial η^2^ = 0.005], Revisits [*F*_(2, 260)_ = 1.77, *p* = 0.172, partial η^2^ = 0.013] and AFD [*F*_(2, 260)_ = 0.28, *p* = 0.756, partial η^2^ = 0.002]. The interaction effects of Group, Task, and AOI in ET and NDT% are illustrated in Figures 3–6.

Figures 3–6 present the interaction between Task and AOI at the different groups: ASD and TD. According to Figures 3–6. This interaction effect indicates that the relationship between task and AOI depends on the group.

For the dependent variables with the statistically significant three-way interaction effect between group, task, and AOI, we now present the simple two-way interactions, main effect of group and pairwise comparisons, where needed, separately. Pairwise comparisons are summarized in Table 2.

**Table 2:** The comparison of the mean ± SD of the eye-tracking measures between ASD (*n* = 23) and TD (*n* = 23).

			**ASD**	**TD**	***Statistics***	
**Eye-tracking measures**	**Task**	**AOI**	**Mean ± SD**	**Mean ± SD**	***p***	***d***
Entry time (ms)	Picture	Faces	1313.07 ± 2526.63	551.51 ± 637.78	0.023^*^	−0.413
		Objects	1685.33 ± 1134.27	2347.37 ± 1967.34	0.049^*^	0.412
	Cartoons	Faces	375.59 ± 375.76	370.32 ± 291.46	0.987	−0.016
		Objects	742.39 ± 410.65	617.82 ± 207.70	0.706	−0.383
	Book	Faces	1120.47 ± 514.29	1124.77 ± 439.73	0.99	0.009
		Objects	3821.01 ± 1311.36	3293.79 ± 708.56	0.116	−0.497
Normalized dwell (ms/coverage)	Picture	Faces	261306.23 ± 200946.10	358870.71 ± 214344.38	0.006^*^	0.47
		Objects	170705.95 ± 122808.11	144670.91 ± 79,944.23	0.457	−0.251
	Cartoons	Faces	373543.36 ± 154277.07	324851.21 ± 107742.37	0.160	−0.366
		Objects	148538.82 ± 75339.15	186859.44 ± 70924.83	0.269	0.524
	Book	Faces	166567.76 ± 82569.63	143424.55 ± 59218.12	0.509	−0.321
		Objects	51705.32 ± 19204.18	51205.75 ± 18,307.33	0.989	−0.027
Net dwell time (%)	Picture	Faces	17.42 ± 7.55	20.95 ± 8.66	0.091	0.435
		Objects	17.86 ± 5.90	15.16 ± 6.70	0.197	−0.428
	Cartoons	Faces	35.59 ± 9.93	36.03 ± 7.95	0.832	0.049
		Objects	30.55 ± 8.06	37.37 ± 7.28	0.001^*^	0.888
	Book	Faces	23.89 ± 6.93	23.53 ± 5.54	0.861	−0.057
		Objects	11.90 ± 3.00	12.98 ± 2.17	0.605	0.411
Fixation time (%)	Picture	Faces	16.62 ± 8.36	20.84 ± 10.35	0.057	0.449
		Objects	16.41 ± 6.16	14.80 ± 7.78	0.469	−0.229
	Cartoons	Faces	34.26 ± 9.85	34.84 ± 8.18	0.792	0.064
		Objects	29.36 ± 8.09	36.24 ± 7.52	0.002^*^	0.881
	Book	Faces	22.87 ± 7.07	22.50 ± 5.75	0.867	−0.057
		Objects	11.30 ± 3.06	12.35 ± 2.25	0.636	0.390

Pairwise comparisons with a Bonferroni adjustment: significance levels and effect-sizes;

* p < 0.05. All comparisons signaled with ^*^ are significant. Effect sizes were computed using Cohen's d.

### Entry Time

Follow-up univariate two-way ANOVAs were run for the dependent variable ET and the main effect of group considered. These showed a statistically significant simple two-way interaction between Group and AOI in the dependent variable Entry Time, for the Picture Task [*F*_(2, 260)_ = 9.08, *p* = 0.003, partial η^2^ = 0.034] but not for the Cartoons [*F*_(2, 260)_ = 0.07, *p* = 0.799, partial η^2^ = 0.000] and Book [*F*_(2, 260)_ = 1.27, *p* = 0.262, partial η^2^ = 0.005] Tasks. As such, a simple main effect analysis was conducted for Picture Task, and we found a statistically significant main effect of Group in the dependent variable Entry Time, for the AOI Faces *F*_(2, 260)_ = 5.194, *p* = 0.023, partial η^2^ = 0.020, and for the AOI Objects *F*_(2, 260)_ = 3.93, *p* = 0.049, partial η^2^ = 0.015, in the Picture Task. Therefore, simple pairwise comparisons were run for the differences in mean ET score in the AOI Faces and AOI Objects between groups in the Picture Task, with a Bonferroni adjustment applied. In the Picture Task, in the AOI Faces, the mean ET in the ASD group was 1313.08 [standard deviation (*SD*) = 2526.63] and 551.51 (*SD* = 637.78) in the TD group, a statistically significant mean difference of 761.56, 95% CI (103.55, 1419.57), *p* = 0.023, *d* = −0.41. In the same task, in the AOI Objects, the mean Entry Time in the ASD group was 1685.34 (*SD* = 1134.27) and 2347.37 (*SD* = 1967.34) in the TD group, a statistically significant mean difference of −662.038, 95% CI (−1320.05, −4.03), *p* = 0.049, *d* = 0.41.

### Normalized Dwell

Follow-up univariate two-way ANOVAs were run for the dependent variable NormD and the main effect of group considered. These showed a statistically significant simple two-way interaction between Group and AOI in the dependent variable NormD, for the Picture task [*F*_(2, 260)_ = 6.25, *p* = 0.013, partial η^2^ = 0.023] but not for the Cartoons [*F*_(2, 260)_ = 3.17, *p* = 0.076, partial η^2^ = 0.012] and Book [*F*_(2, 260)_ = 0.21, *p* = 0.647, partial η^2^ = 0.001] Tasks. Afterwards, a simple main effect analysis was conducted for Picture Task, and we found a statistically significant main effect of Group in the dependent variable NormD, for the AOI Faces *F*_(2, 260)_ = 7.79, *p* = 0.006, partial η^2^ = 0.029. As such, simple pairwise comparisons were run for the differences in mean NormD score in the AOI Faces and AOI Objects between groups in the Picture Task, with a Bonferroni adjustment applied. In the AOI faces in the Picture Task, the mean NormD in the ASD group was 261306.24 (*SD* = 200946.01) and 358870.71 (*SD* = 214344.38) in the TD group, a statistically significant mean difference of −97564.48, 95% CI (−166398.89, −28730.07), *p* = 0.006, *d* = 0.47.

### Net Dwell Time

Follow-up univariate two-way ANOVAs were run for the dependent variable NTD% and the main effect of group considered. These showed a statistically significant simple two-way interaction between Group and AOI in the dependent variable Net Dwell Time, for the task Picture *F*_(2, 260)_ = 4.46, *p* = 0.036, partial η^2^ = 0.017, and for the task Cartoons *F*_(2, 260)_ = 4.80, *p* = 0.029, partial η^2^ = 0.018 but not for the Book [*F*_(2, 260)_ = 0.24, *p* = 0.625, partial η^2^ = 0.001] Task. As such, a simple main effect analysis was conducted for Picture and Cartoons Tasks, and we found a statistically significant main effect of Group in the dependent variable Net Dwell Time, for the AOI Faces in the Cartoons Task *F*_(2, 260)_ = 10.97, *p* = 0.001, partial η^2^ = 0.040. Therefore, simple pairwise comparisons were run for the differences in mean NTD% score in the AOI Faces between groups in the Cartoons Task, with a Bonferroni adjustment applied. In the AOI objects in the Cartoons Task, the mean Net Dwell Time in the ASD group was 30.55 (*SD* = 8.06) and 37.37 (*SD* = 7.28) in the TD group, a statistically significant mean difference of −6.82, 95% CI (−10.88, −2.77), *p* = 0.001, *d* = 0.89.

### Fixation Time

Follow-up univariate two-way ANOVAs were run for the dependent variable FT% and the main effect of group considered. These showed a statistically significant simple two-way interaction between Group and AOI in the dependent variable FT%, for the task Cartoons [*F*_(2, 260)_ = 4.15, *p* = 0.043, partial η^2^ = 0.016]. Afterwards, a simple main effect analysis was conducted for Cartoons Task, and we found a statistically significant main effect of Group in the dependent variable FT%, for the AOI Objects in the Cartoons Task *F*_(2, 260)_ = 9.89, *p* = 0.002, partial η^2^ = 0.037. As such, simple pairwise comparisons were run for the differences in mean FT% score in the AOI Objects between groups in the Cartoons Task, with a Bonferroni adjustment applied. In the AOI objects in the Cartoons Task, the mean Fixation Time in the ASD group was 29.36 (*SD* = 8.09) and 36.24 (*SD* = 7.52) in the TD group, a statistically significant mean difference of −6.88, 95% CI (−11.18, −2.57), *p* = 0.002, *d* = 0.88.

## Discussion

We studied social attention deficits in ASD and focused, in particular, on face stimuli, in the clinical context of different tasks of ADOS. For that purpose, we used eye-tracking methodology to compare the task dependence of visual attention to social stimuli (faces) vs. nonsocial stimuli (objects) in two matched groups of children and adolescents with ASD or TD.

We found significant interaction effects (between group, task, and type of AOI), when the participants are requested to perform spontaneous and simple descriptions of a picture or even a set of cartoons. When scenarios implied generating a goal-oriented narrative in the task, the pattern of attentional allocation in ASD subjects was normalized. In other words, it became similar to controls when children have to create a more complex story, such as the story of a book, doing an enforced narrative description. The absence of interaction effects in that case corroborates similar visual search patterns in TD children. The “Description of a Picture task,” despite being a painting and not a real picture, is the one depicting a more ecological social interaction: a table surrounded by people interacting while having a lunch party, playing guitar, and talking to each other. Our findings thereby provide a framework that reconciles previous literature. Scenes depicting ecological social interactions have been associated to better evoke robust social responses (33, 34). Currently, a methodological approach, denominated cognitive ethology, also focused on ecological validity. It advocates that one's research approach should begin at the level of the phenomenon of interest and to systematically move toward a simplified and abstract level but also doing the inverse approach, reinforcing the idea of analyzing a continuum (49, 50). In our study, we presented three different tasks, with large clinical relevance (used in ADOS) with a growing level of abstract social scenes (from the representation of the social gathering around the table, to the interaction of cartoons in the story of the book).

As predicted, we found that TD children looked first to faces and during a longer period of time in the socially rich and familiar context of a gathering of people around a table (“Description of a Picture task”). The ASD children did not show a differential pattern, between faces and objects. In other words, under these conditions interaction effects are triggered: the ASD group tends to have a similar pattern of visual search in what concerns to attention to social and non-social stimuli, that is, faces and objects, while the TD group looks first at faces and for a longer period of time, which corroborates the hypothesis that ASD participants are less attentive to faces (21–23, 36–39).

On the other hand, our study corroborates that children and adolescents with and without ASD show remarkably similar visual search patterns in their initial eye gaze to faces (51). However, in our study, participants are not instructed to specifically look at faces, which adds meaning and ecological importance to the result. In fact, the participants only had to describe what they were seeing, and therefore, the visual search pattern is natural and more spontaneous.

Overall, our results seem to provide a unifying view of previous research. The TD group always presented a lower ET to the AOI faces than objects, looking at the faces first, when exploring visually the images, also spending more time looking at social stimuli. This visual search pattern is absent in the ASD group. In fact, although children with ASD look at the faces first (lower ET in the AOI faces, than in the AOI objects), there are no statistical differences in the Cartoons and Book tasks (the ones that guide a goal oriented narrative), when compared with TD.

Taken together, our results point to the fact that social attention allocation patterns in ASD population are strongly task dependent, which extends our previous work in other cognitive domains (45). Accordingly, the task not requiring an explicit goal-oriented narrative yields the greatest differences. This raises the question whether spontaneous attention deficits can be rescued by guiding goal-directed actions (40). These results are relevant for the selection of interventional strategies and in ASD children, since it stresses the importance of goal-oriented actions, which are the foundations of the structured teaching.

ASD and TD groups analyze social and non-social content differently. However, when they have to do a narrative, visual behavior tends to normalize (that is, ASD has similar visual patterns as TD), which suggests that the narrative is used as guidance.

Entry time is an eye-tracking measure that characterizes rapid events, and it is in this measure that there are more differences between ASD and TD, which can be interpreted as the best measures to distinguish the groups.

In the present study, there are some limitations to consider. Inclusion of younger children and subjects with intellectual disability was difficult because most of them were not able to make a good calibration and were therefore excluded. Our experimental design did not explicitly account for low-level saliency. However, we believe that, in accordance with previous studies, gaze behavior is better predicted by the social features than by low-level saliency alone (52–54).

In the current analysis, we focused on attention to the faces and objects in the pictures from the different tasks of ADOS (a well-validated but examiner's dependent diagnosis tool for ASD) thereby trying to provide a complementary quantitative information of potential value in clinical practice to distinguish between ASD children without intellectual disability and TD. Although precise, the sensitivity of eye-tracking as a diagnostic tool remains uncertain. Here, we provide evidence for task dependence, with patterns “normalizing” when a narrative is required. With this strategy, we hope to provide a tool that may help improve the course of ASD diagnostic evaluation, especially in subjects with ASD without intellectual disability, where the differential diagnosis with a typical neurodevelopment is often very difficult.

In conclusion, eye-tracking measures of visual scanning, while exploring and describing activities from the ADOS, in particular, “Description of a Picture,” can discriminate between ASD and TD groups. Individuals with ASD allocated their attention to faces and objects in a similar way, while individuals from the TD group attend first and more time to faces. However, when ASD children are asked to look at pictures, organize the thought, and tell a story, they attend to the same stimuli and have a similar pattern of visual search as the TD group, which raises interesting insights on the origin of this “normalization.” Accordingly, when goal-directed actions, in this case, an enforced narrative description, are required, visual search patterns in ASD tend to resemble TD and therefore “normalize” as compared to spontaneous attention. These findings are of potential relevance to training strategies, by providing clues on learning adaptive attentional deployment. In addition, they stress the importance from a diagnostic perspective point of view of observation and classification of spontaneous behavior. Future work should confirm the value of this tool to help differential diagnosis especially in difficult cases with other neurodevelopmental disorders or typical development.

## Data Availability Statement

The raw data supporting the conclusions of this article will be made available by the authors, without undue reservation.

## Ethics Statement

All procedures in this study were reviewed and approved by the ethics committees from the Faculty of Medicine from University of Coimbra, Portugal (CE-11/2013) and the Centro Hospitalar e Universitário de Coimbra, Portugal (CHUC-102-13) and was conducted in accordance with the 1964 Helsinki declaration and its later amendments or comparable ethical standards. Written informed consent was obtained from the parents/guardians of all participants. Children and adolescents also gave oral informed consent.

## Author Contributions

SM, GO, and MC-B: conceived and designed the study. SM: performed the study and wrote the original manuscript. SM, CC, DS, and FD: contributed with data collection. SM and JC: analyzed the data. GO and MC-B: supervision. JC, GO, and MC-B: reviewed and edited the manuscript. All authors read and approved the final manuscript.

## Conflict of Interest

The authors declare that the research was conducted in the absence of any commercial or financial relationships that could be construed as a potential conflict of interest.

## Abbreviations

ADI-R: Autism Diagnostic Interview–Revised
ADOS: Autism Diagnostic Observation Schedule
AFD: Average Fixation Duration
ANOVA: analysis of variance
AOI: area of interest
ASD: autism spectrum disorder
CHUC: Centro Hospitalar E Universitário De Coimbra
CI: confidence interval
DSM: Diagnostic and Statistical Manual of Mental Disorders
DT%: Dwell Time Percentage
ET: Entry Time
FFD: First Fixation Duration
FSIQ: Full-Scale Intelligence Quotient
FT%: Fixation Time Percentage
IQ: Intelligence Quotient
MANOVA: multivariate analysis of variance
NDT%: Net Dwell Time Percentage
NormD: Normalized Dwell
PIQ: Performance IQ
SD: standard deviation
TD: typical neurodevelopment.
